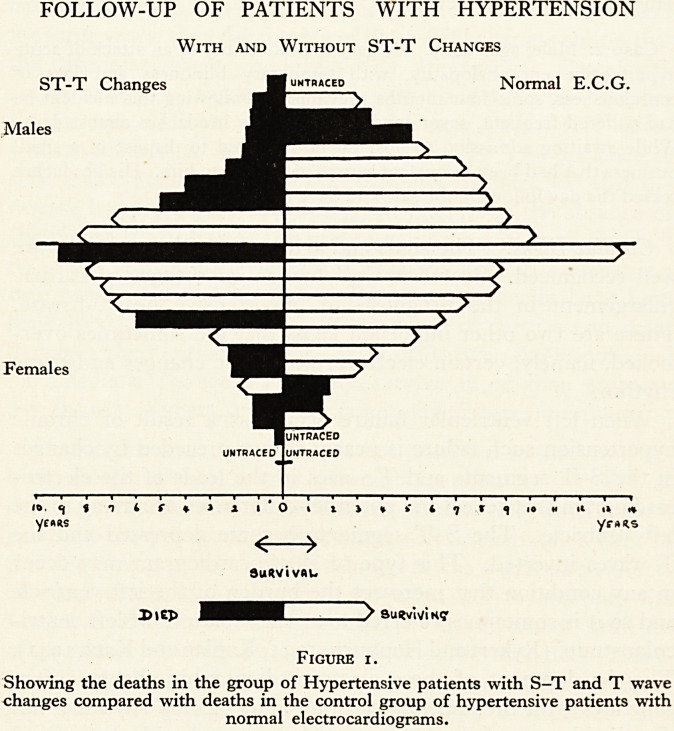# The Prognosis in Hypertension
*Introducing a discussion on Hypertension by the Bristol Medico-Chirurgical Society, on Wednesday, May 10th, 1950.


**Published:** 1950-10

**Authors:** D. H. Davies

**Affiliations:** Physician, United Bristol Hospitals


### The Bristol Medico-Chirurgical Journal

*A Journal of the Medical Sciences for the*

### West of England and South Wales

*"Scire est nescire, nisi id me*
*Scire alius sciret."*

OCTOBER, 1950

### THE PROGNOSIS IN HYPERTENSION*

BY

D. H. DAVIES, M.D., M.R.C.P.
*Physician, United Bristol Hospitals*

In essential hypertension cardiac output, blood volume and blood viscosity are normal, and the rise in blood pressure is due to an increase in the peripheral resistance. Peripheral resistance is largely controlled by the arterioles, and it has been calculated that if the diameter of these vessels is diminished by only one-fifth then the blood pressure is doubled (Wakerlin, 1947). In essential hypertension the initial change is a functional spasm. But later, probably as a result of the increased blood pressure, organic obliterative changes occur: there is an increase in the muscular layer and proliferation and degeneration of the intima. These changes tend to narrow the lumen permanently. In the benign form of the disease these arteriolar changes usually progress very slowly: that is why, even after many years, it is rare for the changes in such organs as the kidney to have advanced to a degree sufficient to interfere with function. However, it sometimes happens that the tempo of the arteriolar disease is either rapid from the onset or suddenly increases and the organic

* Introducing a discussion on Hypertension by the Bristol Medico-Chirurgical Society, on Wednesday, May 10th, 1950.

changes in the vessels advance to the stage of necrosis. It is this arteriolar necrosis which is the characteristic pathological lesion of the malignant form of the disease, the clinical features of which are a rapidly progressive downhill course, a high fixed blood pressure and the presence of papilloedema.

There are surprisingly few satisfactory follow-up studies of essential hypertension in the literature. Many do not separate the malignant cases and others include hospital in-patients already in heart failure. There are, however, two reports that give a fairly good perspective of the disease in ambulant subjects: namely those of Burgess and Bechgaard.

Burgess (1948) followed up one hundred cases of uncomplicated essential hypertension: that is to say, patients with hypertension but with little or no evidence of heart disease, with good renal function and with fundi that showed only the early changes of hypertensive disease. He showed that this type of patient usually lives to within three or four years of his normal expectation of life: except in the younger age groups, where he found a significant increase in mortality. When considering these results it is important to remember that of Burgess's hundred cases, eighty were females. For it is generally agreed that the prognosis is better in women than in men.

Bechgaard (1946) followed a very large series of ambulant cases, using as a control the mortality rate of the general population of Denmark during the period of his study. He found that the mortality in males was twice that in females; in the latter it was only slightly greater than normal, when the diastolic blood pressure was less than 130 mm. Hg. In both sexes the highest mortality occurred when hypertension developed before the sixth decade and it was particularly high among young males. After the sixth decade the mortality rate decreased very appreciably with age. Heart disease was another factor associated with a high death rate. Seventy-eight per cent. of males and 49 per cent. of females who showed evidence of heart disease when first examined died within two years.

### Assessment of an Individual Case

*The Blood Pressure.* When attempting to assess an individual case it must be remembered that essential hypertension is characterized not only by a raised blood pressure but also by an exaggerated blood-pressure-response to all manner of pressor stimuli, such as emotion. The blood pressure is continually varying and the swing in both systolic and diastolic readings may fluctuate as much as 30 per cent. on either side of the average mean figure (Kilpatrick). Thus when a considerable rise or fall in pressure is recorded care must be taken before attributing this change to any deterioration or improvement in the disease.

*Coronary Disease.* It is difficult to discuss essential hypertension without some reference to coronary disease. For although hypertension is not the cause of arteriosclerosis, there is no doubt that a raised blood pressure accelerates such arterial changes. The necropsy study of Davis and Klainer (1940) showed that the presence of hypertension greatly increases the incidence of coronary atheroma in both males and females. In hypertensive males under the age of fifty they found evidence of coronary atheroma as extensive as that in males up to and over the age of seventy. In females under fifty who had had normal blood pressure there was very little disease, but the incidence became significant when hypertension had been present. This last finding re-affirms the teaching that one should hesitate to diagnose cardiac pain in a female less than fifty years of age in the absence of a raised blood pressure, unless there is an associated lesion such as aortic disease or anaemia.

*The Fundi.* The occular fundus is the one place where the changes in the arterioles can be easily seen. Moreover, these vessels reflect, within certain limits, the changes that are occurring in the arterioles throughout the body. Careful examination of the fundi is therefore essential when attempting to assess the severity of the vascular disease, and repeated examinations over a period of time give valuable information about the rate of progress.

Wagner and Keith (1939) have suggested a very useful classification of the hypertensive arteriolar changes seen in the fundi. They have divided them into four grades of severity and this grading is now in general use. Briefly, Grades I and II consist of those cases which show slight narrowing of the lumen as a result of spasm, and thickening of the vessel wall without retinitis. Grades I and II differ only in degree and the majority of cases of essential hypertension fall into one or other of these two groups; and if hypertension is known to have been present for some years, then the finding of these mild changes shows that the arteriolar disease is only slowly progressive. Cases with severe arteriolar spasm together with exudates and haemorrhages, but without papilloedema, fall into Grade III; those with papilloedema in addition into Grade IV. The presence of severe vaso-spastic retinitis with papilloedema signifies that the disease has passed into the malignant phase. In the absence of significant heart disease the prognosis of patients with Grade I or Grade II arteriolar changes is usually good and uraemia is a rare event. Cases with Grade III changes usually have high fixed blood pressures, and often evidence of cardiac and renal damage: their outlook is poor. While those who show additional papilloedema (Grade IV) rarely survive more than eighteen months to two years, and death from renal failure is common.

*Central Nervous System.* It is not proposed to discuss cerebral vascular accidents, which account for about 15 per cent. of deaths from essential hypertension. But a few words about headache are pertinent as the subject will be mentioned later in the discussion, particularly in relation to the therapeutic effects of sympathectomy. Care must be taken not to attribute the headache of a hypertensive subject to hypertension until all other causes of headache have been carefully excluded. Headache is a common symptom and is often encountered in anxiety and nervous ill-health. It is therefore not unexpected that simple "anxiety" headaches are frequently found in hypertensive patients already aware of their increased blood pressure. It is sometimes very difficult to distinguish such headaches from those directly due to hypertension, as in the following two cases:

**Case 1.** Female, aged forty-eight. She was first seen ten months ago. She gave a history of severe headache, which had been present for eight years. The headaches occurred on waking in the morning, tending to wear off during the day. They were becoming more frequent and occurred almost daily. She was seen with a view to sympathectomy. Examination revealed a blood pressure of 230/130, but no other significant abnormality; radiologically the heart was practically normal in size and the electrocardiogram was satisfactory; her fundi showed only early changes (Grade I). Her blood pressure fell considerably with rest and it was felt that her prognosis was good. She was reassured and discharged from hospital and had since remained symptom-free, although her blood pressure has returned to its original level.

**Case 2.** Male, aged sixty. He gave a clear history of an attack of acute hypertensive encephalopathy, with temporary blindness and loss of consciousness, some four months previously. Following this incident he had suffered frequent, severe and incapacitating headaches almost daily. While awaiting admission to hospital he managed to dispose of a small business that had been a constant source of anxiety to him. His headaches ceased the day following the sale and have not recurred.

*Cardiac Signs.* The signs and symptoms of heart failure are well recognized. So too is the obvious importance of cardiac enlargement in the prognosis of hypertensive heart disease. There are two other important signs that are sometimes overlooked, namely, certain electrocardiographic changes and triple rhythm.

When left ventricular failure occurs as a result of chronic hypertension such failure is nearly always preceded by changes in the S–T segments and T waves in the leads of the electrocardiogram that reflect the potential-differences occurring in the left ventricle. The S–T segments become depressed and the T waves inverted. This type of electrocardiogram may occur in any condition that increases the burden of the left ventricle and so it is sometimes referred to as the " pattern of left ventricular strain" (Rykert and Hepburn, 1934; Kaplan and Katz, 1941). The development of these electrocardiographic changes often coincides with the appearance of an apical gallop rhythm: but the significance of the latter must be assessed with due regard to heart rate and possible defects in auriculo-ventricular conduction. Fig. 1 shows the mortality of a small group of hypertensive patients put at the disposal of Dr. David Short by Professor Perry. The group of patients with electrocardiographic changes was matched with a control group with respect to age, sex and blood pressure. All cases with coronary disease, aortic disease and any other condition that might have influenced the electrocardiogram were carefully excluded from both groups. It was unfortunately impossible to ascertain the mode of death in most of the cases: but the figure shows a considerably higher mortality in the group with the electrocardiographic changes than in the control.

FOLLOW-UP OF PATIENTS WITH HYPERTENSION

With and Without ST-T Changes

Figure 1.

Showing the deaths in the group of Hypertensive patients with S–T and T wave changes compared with deaths in the control group of hypertensive patients with normal electrocardiograms.

Cardiac enlargement together with gallop rhythm, and S–T and T wave changes in the electrocardiogram may be found in the absence of cardiac symptoms. But these signs when present change the diagnosis from uncomplicated Essential Hypertension to that of Hypertensive Heart Disease, and they must not be overlooked, therefore, when assessing prognosis.

### Summary

1. Essential hypertension is often compatible with a full and useful life, and with an expectation that is not far short of normal.

2. The prognosis is better in women than men.

3. The outlook is less satisfactory in young subjects, especially young males.

4. If we have a procedure that will reduce the blood pressure satisfactorily in essential hypertension then it is suggested that the most deserving cases are the young patients, especially the young males: particularly those who show changes in their fundi indicative of severe arteriolar disease or changes that are progressive and those who have early and not irreparable cardiac damage. In addition there are the cases who have passed into the malignant phase of the disease, provided the renal function is satisfactory.

### REFERENCES

Bechgaard, P. (1946).—*Acta Med. Scand. Supplement*, 172.
Burgess, A. M. (1948).—*New Eng. J. Med.*, **239**, 75.
Davis, D., and Klainer, M. (1940).—*Am. Heart J.*, **19**, 185.
Kaplan, L. G., and Katz, L. N. (1941).—*Amer. J. Med. Sci.*, **201**, 676.
Kilpatrick, J. A. (1948).—*Brit. Heart J.*, **10**, 48.
Rykert, H. E., and Hepburn, J. (1934–35).—*Am. Heart J.*, **10**, 942.
Wagner, H., and Keith, N. M. (1939).—*Medicine*, **18**, 317.
Wakerlin, G. E. (1949).—*Ann. Int. Med.*, **31**, 312.

## Figures and Tables

**Figure 1. f1:**